# Headache and psychopathology: DSM-V vs ICHD-3β vs ICD10

**DOI:** 10.1186/1129-2377-16-S1-A41

**Published:** 2015-09-28

**Authors:** Maurizio Pompili

**Affiliations:** Department of Neurosciences, Mental Health and Sensory Organs, Suicide Prevention Center, Sant'Andrea Hospital, Sapienza University of Rome, Rome, Italy

A strict relationship between migraine and psychiatric factors has been suggested, but the exact role and the influence on evolution of headache is unknown. The most frequent diagnosis was a comorbidity of anxiety and mood disorders. The comorbidity of psychiatric disorders and headache has important implications as far as treatment is concerned.

In comparing DSM-V, ICHD-3β and ICD10 criteria on headache and psychopathology, diagnostic criteria agree on pre-existing headache with the characteristics of a primary headache disorder becoming chronic, or made significantly worse, in close temporal relation to a psychiatric disorder, both in the initial headache diagnosis and psychiatric diagnosis. Headache attributed to psychiatric disorder should be given, provided that there is good evidence that that disorder can cause headache. When a causal relationship cannot be confirmed, the pre-existing primary headache and the psychiatric disorder are diagnosed separately. Thus, the diagnostic categories are limited to those few cases in which a headache occurs in the context and as a direct consequence of a psychiatric condition known to be symptomatically manifested by headache. Diagnostic criteria must be restrictive enough not to include false positive cases, but must set the threshold low enough to admit the majority of affected patients. Headache disorders occur coincidentally with a number of psychiatric disorders. Although criteria for headaches attributed to psychiatric disorders have suggested that headache occurring exclusively in association with several common psychiatric disorders, such as, depressive, anxiety and trauma/stress-related disorders, might be considered as attributed to these disorders, because of uncertainties concerning the causal relationships and relative lack of evidence in this context. Evidence suggests that the presence of a comorbid psychiatric disorder tends to worsen the course of migraine and/or tension-type headache by increasing the frequency and severity of the headache and/or making it less responsive to treatment. Thus, identification and treatment of any comorbid psychiatric condition is important for the proper management of these headaches. It should be noted that somatization disorder per se is not included in the fifth edition of the Diagnostic and Statistical Manual of Mental Disorders (DSM-V), it has been replaced by the category Somatic Symptom Disorder, characterized by one or more somatic symptoms. Thus, ICHD-3 beta continues to refer to the DSM-IV definition of somatization disorder. Using WHO's criteria and methods for measuring burden of disease in DALYs, headache disorders can be placed correctly in the context of other mental and neurological disorders and other chronic illnesses. In order to know the full burden attributable to headache disorders, however, further epidemiological work must be conducted around the world and this must encompass assessments of clinical, economic and humanistic impacts.

